# Endovascular therapy in acute ischaemic stroke patients with prestroke dependency—an EVA-TRISP cohort study

**DOI:** 10.1093/esj/aakag099

**Published:** 2026-08-21

**Authors:** Ines Piot, Lukas S Enz, Valerian L Altersberger, Simon Trüssel, Gian Marco De Marchis, Tolga Dittrich, Mirjam R Heldner, Julian F Hotz, Greta Soekeland, Tomas Dobrocky, Alessandro Pezzini, Patrik Michel, Davide Strambo, Alexander Salerno, Susanne Wegener, Corinne Inauen, Philipp Baumgartner, Sami Curtze, Nicolas Martinez-Majander, Paul J Nederkoorn, Jonathan M Coutinho, Christian H Nolte, Christoph Riegler, Visnja Padjen, Ronen R Leker, Hamza Jubran, Malin Woock, Katarina Jood, Alexandros Rentzos, Carlo W Cereda, Marco Vabanesi, Andrea Zini, Matteo Paolucci, João Pedro Marto, Alexandra Martins, Jeremy Molad, Stefan T Engelter, Henrik Gensicke, for the EndoVAscular treatment and Thrombolysis for Ischemic Stroke Patients (EVA-TRISP) collaborators

**Affiliations:** Department of Neurology and Stroke Center, University Hospital Basel, Basel, Switzerland; Department of Clinical Research, University Hospital Basel and University of Basel, Basel, Switzerland; Department of Neurology and Stroke Center, University Hospital Basel, Basel, Switzerland; Department of Clinical Research, University Hospital Basel and University of Basel, Basel, Switzerland; Department of Neurology and Stroke Center, University Hospital Basel, Basel, Switzerland; Department of Clinical Research, University Hospital Basel and University of Basel, Basel, Switzerland; Department of Neurology and Stroke Center, University Hospital Basel, Basel, Switzerland; Department of Clinical Research, University Hospital Basel and University of Basel, Basel, Switzerland; Department of Neurology and Neurorehabilitation, University Department of Geriatric Medicine Felix Platter, University of Basel, Basel, Switzerland; Department of Clinical Research, University Hospital Basel and University of Basel, Basel, Switzerland; Department of Neurology and Stroke Center, University Teaching and Research Hospital, Kantonsspital St. Gallen, St. Gallen, Switzerland; Department of Clinical Research, University Hospital Basel and University of Basel, Basel, Switzerland; Department of Neurology and Stroke Center, University Teaching and Research Hospital, Kantonsspital St. Gallen, St. Gallen, Switzerland; Department of Neurology and University Institute of Diagnostic and Interventional Neuroradiology, Inselspital, Bern University Hospital, University of Bern, Bern, Switzerland; Department of Neurology and University Institute of Diagnostic and Interventional Neuroradiology, Inselspital, Bern University Hospital, University of Bern, Bern, Switzerland; Department of Neurology and University Institute of Diagnostic and Interventional Neuroradiology, Inselspital, Bern University Hospital, University of Bern, Bern, Switzerland; Department of Neurology and University Institute of Diagnostic and Interventional Neuroradiology, Inselspital, Bern University Hospital, University of Bern, Bern, Switzerland; Department of Medicine and Surgery, University of Parma, Parma, Italy; Stroke Care Program, Department of Emergencies, Parma University Hospital, Parma, Italy; Stroke Center, Service of Neurology, Lausanne University Hospital and University of Lausanne, Lausanne, Switzerland; Stroke Center, Service of Neurology, Lausanne University Hospital and University of Lausanne, Lausanne, Switzerland; Stroke Center, Service of Neurology, Lausanne University Hospital and University of Lausanne, Lausanne, Switzerland; Department of Neurology, University Hospital Zurich and University of Zurich, Zurich, Switzerland; Department of Neurology, University Hospital Zurich and University of Zurich, Zurich, Switzerland; Department of Neurology, University Hospital Zurich and University of Zurich, Zurich, Switzerland; Department of Neurology, Helsinki University Hospital and University of Helsinki, Helsinki, Finland; Department of Neurology, Helsinki University Hospital and University of Helsinki, Helsinki, Finland; Department of Neurology, Amsterdam UMC Location University of Amsterdam, Amsterdam, The Netherlands; Department of Neurology, Amsterdam UMC Location University of Amsterdam, Amsterdam, The Netherlands; Department of Neurology with Experimental Neurology, Charité - Universitätsmedizin Berlin, corporate member of Freie Universität Berlin and Humboldt-Universität zu Berlin, Berlin, Germany; Center for Stroke Research Berlin (CSB), Charité - Universitätsmedizin Berlin, Berlin, Germany; Department of Neurology with Experimental Neurology, Charité - Universitätsmedizin Berlin, corporate member of Freie Universität Berlin and Humboldt-Universität zu Berlin, Berlin, Germany; Center for Stroke Research Berlin (CSB), Charité - Universitätsmedizin Berlin, Berlin, Germany; Neurology Clinic, University Clinical Centre of Serbia, and Faculty of Medicine, University of Belgrade, Belgrade, Serbia; Department of Neurology, Hadassah-Hebrew University Medical Center, Jerusalem, Israel; Department of Neurology, Hadassah-Hebrew University Medical Center, Jerusalem, Israel; Department of Neurology, Sahlgrenska University Hospital, Gothenburg, Sweden; Department of Clinical Neuroscience, Institute of Neurosciences and Physiology, Sahlgrenska Academy, University of Gothenburg, Gothenburg, Sweden; Department of Neurology, Sahlgrenska University Hospital, Gothenburg, Sweden; Department of Clinical Neuroscience, Institute of Neurosciences and Physiology, Sahlgrenska Academy, University of Gothenburg, Gothenburg, Sweden; Department of Radiology, Institute of Clinical Sciences, Sahlgrenska Academy, University of Gothenburg, Gothenburg, Sweden; Department of Radiology, Section of Diagnostic and Interventional Neuroradiology, Sahlgrenska University Hospital, Region Västra Götaland, Gothenburg, Sweden; Stroke Center, Department of Neurology, Neurocenter of Southern Switzerland, Ente Ospedaliero Cantonale (EOC), Lugano, Switzerland; Stroke Center, Department of Neurology, Neurocenter of Southern Switzerland, Ente Ospedaliero Cantonale (EOC), Lugano, Switzerland; Department of Neurology and Stroke Center, IRCCS Istituto delle Scienze Neurologiche di Bologna, Maggiore Hospital, Bologna, Italy; Department of Neurology and Stroke Center, IRCCS Istituto delle Scienze Neurologiche di Bologna, Maggiore Hospital, Bologna, Italy; Department of Neurology, Hospital de Egas Moniz, ULS Lisboa Ocidental, Lisbon, Portugal; Lisbon Clinical Academic Center, NOVA Medical School, Universidade NOVA de Lisboa, Lisbon, Portugal; Department of Neurology, Hospital de Egas Moniz, ULS Lisboa Ocidental, Lisbon, Portugal; Department of Stroke & Neurology, Tel-Aviv Sourasky Medical Center, Tel-Aviv, Israel; Gray Faculty of Medicine, Tel-Aviv University, Tel-Aviv, Israel; Department of Neurology and Stroke Center, University Hospital Basel, Basel, Switzerland; Department of Clinical Research, University Hospital Basel and University of Basel, Basel, Switzerland; Department of Neurology and Neurorehabilitation, University Department of Geriatric Medicine Felix Platter, University of Basel, Basel, Switzerland; Department of Clinical Research, University Hospital Basel and University of Basel, Basel, Switzerland; Department of Neurology and Neurorehabilitation, University Department of Geriatric Medicine Felix Platter, University of Basel, Basel, Switzerland

**Keywords:** functional outcome, mechanical thrombectomy success, mortality, mRS, real-world registry, sICH

## Abstract

**Introduction:**

Acute EVT may be withheld from stroke patients with prestroke dependency on the help of others in daily activities because of insufficient randomised evidence. Using data from a large registry, we aimed to improve evidence for EVT decision-making in these patients.

**Patients and methods:**

Multicentre international observational study from the EndoVascular Therapy and ThRombolysis in Ischemic Stroke Patients (EVA-TRISP) collaboration comparing prestroke-dependent (prestroke mRS 3–4) with prestroke-independent patients (prestroke mRS 0–2). We used multivariate logistic regression analysis and report odds ratios (ORs) and 95% CIs. Outcomes were poor functional outcome in survivors (defined relative to the prestroke mRS), 3 month mortality, sICH occurrence and unsuccessful recanalisation.

**Results:**

Of 13,525 eligible patients, 1436 (10.6%) were prestroke-dependent. These patients were older (median 84 vs 73 years), more often women (61.0% vs 46.7%) and had higher NIHSS score (median 16 vs 14 points). While the odds of death (OR_adjusted_ 2.33 [95% CI, 2.04–2.66]; 48.6% vs 19.6%) and unsuccessful recanalisation (OR_adjusted_ 1.16 [95% CI, 1.02–1.33]; 31.0% vs 26.6%) were higher in prestroke-dependent patients, there was no evidence of increased odds of poor functional outcome in survivors (OR_adjusted_ 0.68 [95% CI, 0.57–0.81]; 42.8% vs 40.0%) and sICH (OR_adjusted_ 0.72 [95% CI, 0.53–0.98]; 3.8% vs 4.6%).

**Discussion and conclusions:**

Despite higher mortality and recanalisation failure, prestroke-dependent patients did not have worse functional outcomes among survivors or higher sICH risk. This suggests that EVT should not be withheld solely on the basis of prestroke dependency.

## Graphical Abstract

**Graphical Abstract:**
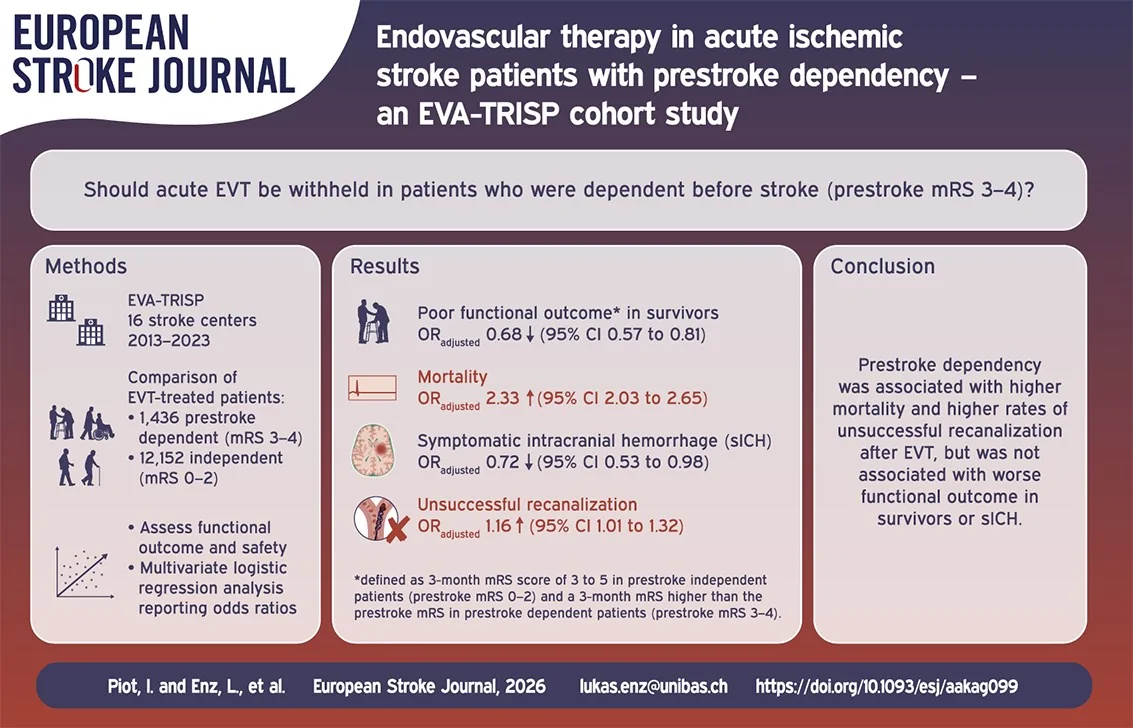

## Introduction

Endovascular treatment (EVT)—with or without intravenous thrombolysis (IVT)—is the established treatment for acute ischaemic stroke caused by large vessel occlusion.^1^^,^^2^

The global trend towards an ageing society and rising cardiovascular burden is leading to an increasing number of acute stroke patients with pre-existing dependency on the help of others in daily activities. Studies suggest that more than one-third of stroke patients have a pre-existing disability.^3^^,^^4^

Randomised controlled trials of EVT recruited few to no patients with prestroke dependency, defined by a prestroke mRS of 3–5.^5–8^ Endovascular treatment may be withheld from this population due to concerns regarding worse functional outcomes, higher complication rates and mortality. Efforts to evolve treatment paradigms for this vulnerable group could influence clinical outcomes as well as the wider health-economic burden.

The need for more evidence has been recognised, and several observational studies^9–17^ as well as meta-analyses^18–21^ have attempted to address this issue. However, these investigations are limited by small sample sizes, substantial rates of missing follow-up data and single-centre or single-country design. Several studies were also conducted during periods of rapidly evolving EVT techniques and treatment paradigms, which may affect comparability across cohorts. There is a lack of information on the reasons for prestroke dependency and on patients with more severe baseline disability.

To address this gap in evidence, we evaluated the safety of EVT in prestroke-dependent patients using data from an international, real-world, long-term registry: the EndoVascular Therapy and ThRombolysis in Ischemic Stroke Patients (EVA-TRISP) collaboration.^22^^,^^23^

## Patients and methods

For this cohort study, we used prospectively collected data from 16 centres of the international, multicentre EVA-TRISP collaboration (Table S1), which has been described previously.^22^^,^^23^ EndoVascular Therapy-TRISP is an investigator-initiated academic collaboration of comprehensive stroke centres that prospectively collect clinical and imaging data using harmonised variable definitions. Data collection, imaging interpretation and outcome assessment are performed locally at each participating centre. No central imaging core laboratory or external audit process was used for the present study.

We included all patients treated with EVT between January 2015 and June 2023. As previously described,^24^ data for the following baseline variables were included: (1) demographic data (prestroke mRS, age, sex), (2) stroke and treatment characteristics (NIHSS at admission, territory of stroke [anterior, posterior or both], initial ASPECT score,^25^ time from onset to groin or, if unknown time from last seen well to groin, bridging therapy, complications during EVT, intra-arterial thrombolysis, mechanical treatment, general anaesthesia, number of passes, early stopping of procedure), (3) admission vital parameters and laboratory results (systolic blood pressure, glucose level), (4) cardio- and cerebrovascular risk factors (prior ischaemic stroke, coronary artery disease, atrial fibrillation, diabetes mellitus, arterial hypertension, hypercholesterolemia, current smoking or stepped < 2 years) and prior antithrombotic therapy (antiplatelet and oral anticoagulation) and (5) stroke aetiology according to Trial of Org 10172 in Acute Stroke Treatment (TOAST) criteria^26^ (Table S1).

The outcome measures were (1) poor functional outcome in survivors 3 months after stroke, (2) death within 3 months after stroke, (3) sICH and (4) unsuccessful recanalisation. As a secondary outcome, we investigated any ICH (aICH). Poor functional outcome in survivors was defined by a 3-month mRS score of 3–5 in prestroke-independent patients (prestroke mRS 0–2) and a 3-month mRS higher than the prestroke mRS in prestroke-dependent patients (prestroke mRS 3–4) as done previously.^27^^,^^28^ The mRS score after stroke was assessed after 3 months via outpatient consultations or telephone calls with patients and/or relatives. Death was assessed during hospital stay and at 3-month follow-up. Symptomatic ICH was defined using the European Cooperative Acute Stroke Study (ECASS) II criteria as extravascular intracranial blood on imaging combined with clinical worsening (an increase in NIHSS of more than 4 points). If ECASS II was not available, the ECASS III criteria were used (additionally to ECASS II, the bleeding needs to be the suspected cause of the clinical worsening).^29^^,^^30^ Monitoring for intracranial haemorrhage was performed using follow-up CT or MRI.^22^ Follow-up imaging was generally obtained approximately 24 h after treatment or earlier in cases of clinical worsening; however, some centres performed follow-up imaging only when clinical deterioration occurred. Unsuccessful recanalisation was defined as modified thrombolysis in cerebral infarction (mTICI) scores 0–2a^31^ and was assessed by digital subtraction angiography at the end of EVT.

The prestroke mRS score^32^ was prospectively documented at each participating centre as part of routine stroke care, based on information obtained from patients, relatives, caregivers and available medical records. The comparison cohorts were defined as prestroke-independent (prestroke mRS 0–2) and prestroke-dependent patients (prestroke mRS 3–4).

### Putative contributing conditions to prestroke dependency

Potential comorbidities which could have contributed to prestroke dependency were retrospectively gathered through review of the patients’ medical records as done previously.^27^ Categories were previous ischaemic stroke, dementia, Parkinson’s disease, epilepsy, other neurological diseases, internal diseases, active oncological diseases, psychiatric diseases and traumatic or degenerative bone diseases.

### Descriptive data analysis

Continuous data were summarised using the median and IQR; binary data were summarised as percentages. The Mann–Whitney *U* test was used to compare continuous variables and the χ^2^ test was used for categorical data. Both raw and adjusted *P*-values (Bonferroni’s method) are reported. No formal *P*-value cut-off was defined to assume statistical significance, instead effect sizes and *P*-values are discussed.

### Patient exclusion criteria

Patients from the full cohort were excluded from the analysis set if (1) they had a missing prestroke mRS, (2) the prestroke mRS was 5 (because these patients could not achieve a poor outcome if they survived due to the definition), (3) no main outcomes were reported, (4) stroke mimics and (5) patients under the age of 18 years.

### Data cleaning and missing data imputation

We performed limited data cleaning by overwriting logically impossible values and extremely implausible values that hindered model convergence as missing values (“NA”), as reported in the results section.

The absolute and relative numbers of missing data are reported for all baseline characteristics and outcome variables. To minimise the bias caused by a complete case analysis, we performed a multiple imputation by chained equations imputation on the dataset containing all variables reported in Table 1. We followed a “multiple imputation, then delete” approach, thus we also used the rows where one or more of the 4 outcomes was missing for imputation, but we did not use these rows for the analysis of the missing outcomes, ie, the analysis was performed only on reported and not on imputed outcomes. This approach is robust against poor imputation of the outcome and tends to reduce variability in the analysis.^33^ We imputed 100 datasets with 30 iterations per dataset. Numerical data were imputed by predictive mean matching, binary outcomes by logistic regression. All models were calculated on each imputed dataset and the results were pooled according to Rubin’s rules.^34^

**Table 1:** Patient characteristics.

Variable	All (*n* = 13,525)	Prestroke mRS 0–2 (*n* = 12,089)	Prestroke mRS 3–4 (*n* = 1,436)	Raw *P*-value	Adj. *P*-value	Missing data
***Demographics***						
** Prestroke mRS, median (IQR)**	0 (0–1)	0 (0–1)	3 (3–3)	<.001	<.001	0 (0%)/0 (0%)
** Age, median (IQR)**	75 (64–83)	73 (63–81)	84 (76–89)	<.001	<.001	0 (0%)/0 (0%)
** Sex (M)**	6,999/13,512 (51.8%)	6,440/12,078 (53.3%)	559/1,434 (39%)	<.001	<.001	11 (0.1%)/2 (0.1%)
***Stroke characteristics and treatment***						
** NIHSS admission [0–42], median (IQR)**	14 (9–19)	14 (8–19)	16 (11–21)	<.001	<.001	164 (1.4%)/15 (1%)
** Stroke all territories**						2,793 (23.1%)/410 (28.6%)
** Stroke in anterior territory only**	9,077/10,322 (87.9%)	8,157/9,296 (87.8%)	920/1,026 (89.7%)	.081	1	
** Stroke in posterior territory only**	954/10,322 (9.2%)	874/9,296 (9.4%)	80/1,026 (7.8%)	.104	1	
** Stroke in both territories**	291/10,322 (2.8%)	265/9,296 (2.9%)	26/1,026 (2.5%)	.63	1	
** ASPECTS, median (IQR)**	9 (8–10)	9 (8–10)	9 (7–10)	.008	.306	6,281 (52%)/745 (51.9%)
** Time to groin, median (IQR)**	206 (150–310)	205 (150–310)	211 (155–315.2)	.118	1	1,006 (8.3%)/144 (10%)
** Bridging therapy**	6,369/13,513 (47.1%)	5,874/12,080 (48.6%)	495/1,433 (34.5%)	<.001	<.001	9 (0.1%)/3 (0.2%)
** EVT complications**	1,541/9,074 (17%)	1,360/8,184 (16.6%)	181/890 (20.3%)	.006	.226	3,905 (32.3%)/546 (38%)
** Intra-arterial thrombolysis**	618/11,811 (5.2%)	553/10,581 (5.2%)	65/1,230 (5.3%)	.985	1	1,508 (12.5%)/206 (14.3%)
** EVT mechanical thrombectomy**	11,962/13,294 (90%)	10,715/11,872 (90.2%)	1,247/1,422 (87.7%)	.003	.108	217 (1.8%)/14 (1%)
** EVT general anaesthesia**	8,006/12,437 (64.4%)	7,077/11,136 (63.5%)	929/1,301 (71.4%)	<.001	<.001	953 (7.9%)/135 (9.4%)
** EVT number of passes, median (IQR)**	1 (1–3)	1 (1–3)	2 (1–3)	.232	1	9,025 (74.7%)/1,152 (80.2%)
** EVT stopped early**	1,138/9,500 (12%)	975/8,392 (11.6%)	163/1,108 (14.7%)	.003	.132	3,697 (30.6%)/328 (22.8%)
***Vital signs and laboratory results at admission***						
** Admission systolic blood pressure [mmHg], median (IQR)**	150 (131–170)	150 (132–169)	148 (130–170)	.161	1	903 (7.5%)/137 (9.5%)
** Admission glucose [mmol/l], median (IQR)**	6.9 (5.9–8.3)	6.9 (5.9–8.3)	6.9 (5.9–8.4)	.387	1	1,236 (10.2%)/186 (13%)
***Medical history***						
** Prior ischaemic stroke**	1,870/12,807 (14.6%)	1,513/11,480 (13.2%)	357/1,327 (26.9%)	<.001	<.001	609 (5%)/109 (7.6%)
** Coronary artery disease**	2,434/12,620 (19.3%)	2,095/11,325 (18.5%)	339/1,295 (26.2%)	<.001	<.001	764 (6.3%)/141 (9.8%)
** Atrial fibrillation**	4,983/13,457 (37%)	4,207/12,027 (35%)	776/1,430 (54.3%)	<.001	<.001	62 (0.5%)/6 (0.4%)
** Diabetes mellitus**	2,631/13,463 (19.5%)	2,263/12,032 (18.8%)	368/1,431 (25.7%)	<.001	<.001	57 (0.5%)/5 (0.3%)
** Arterial hypertension**	9,296/13,459 (69.1%)	8,161/12,028 (67.8%)	1,135/1,431 (79.3%)	<.001	<.001	61 (0.5%)/5 (0.3%)
** Hypercholesterolaemia**	6,689/13,456 (49.7%)	5,959/12,025 (49.6%)	730/1,431 (51%)	.31	1	64 (0.5%)/5 (0.3%)
** Current smoking or stopped < 2 years**	2,733/13,417 (20.4%)	2,559/11,993 (21.3%)	174/1,424 (12.2%)	<.001	<.001	96 (0.8%)/12 (0.8%)
** Prior platelet inhibition**	3,833/13,352 (28.7%)	3,321/11,937 (27.8%)	512/1,415 (36.2%)	<.001	<.001	152 (1.3%)/21 (1.5%)
** Prior oral anticoagulation**	2,540/12,857 (19.8%)	2,141/11,469 (18.7%)	399/1,388 (28.8%)	<.001	<.001	620 (5.1%)/48 (3.3%)
***Stroke aetiology according to TOAST criteria***						
** TOAST classification**						717 (5.9%)/54 (3.8%)
** Large artery atherosclerosis**	2,593/12,799 (20.3%)	2,331/11,414 (20.4%)	262/1,385 (18.9%)	.2	1	
** Cardioembolic stroke**	5,601/12,799 (43.8%)	4,878/11,414 (42.7%)	723/1,385 (52.2%)	<.001	<.001	
** Stroke of other determined cause**	918/12,799 (7.2%)	873/11,414 (7.7%)	45/1,385 (3.2%)	<.001	<.001	
** More than one**	971/12,799 (7.6%)	852/11,414 (7.5%)	119/1,385 (8.6%)	.149	1	
** Stroke of undetermined cause**	2,671/12,799 (20.9%)	2,438/11,414 (21.4%)	233/1,385 (16.8%)	<.001	.004	
***Outcomes***						
** Poor outcome at 3 months in survivors**	3,926/9,773 (40.2%)	3,632/9,086 (40%)	294/687 (42.8%)	.157	1	0 (0%)/0 (0%)
** Death at 3 months**	2,864/12,637 (22.7%)	2,215/11,301 (19.6%)	649/1,336 (48.6%)	<.001	<.001	788 (6.5%)/100 (7%)
** Symptomatic intracranial haemorrhage**	575/12,656 (4.5%)	524/11,312 (4.6%)	51/1,344 (3.8%)	.185	1	777 (6.4%)/92 (6.4%)
** Any intracranial haemorrhage**	3,074/11,488 (26.8%)	2,753/10,244 (26.9%)	321/1,244 (25.8%)	.44	1	1,845 (15.3%)/192 (13.4%)
** Unsuccessful recanalisation**	3,136/11,571 (27.1%)	2,738/10,286 (26.6%)	398/1,285 (31%)	.001	.041	1,803 (14.9%)/151 (10.5%)

Abbreviations: ASPECTS, Alberta Stroke Program Early Computed Tomography Score; TOAST, Trial of Org 10172 in Acute Stroke Treatment.

The Mann–Whitney *U* test was used to compare continuous variables and χ^2^ test was used for categorical data. Adjusted *P*-values are derived from Bonferronis correction applied across the complete table. Missing data are given per group (previously independent vs dependent) separately.

### Statistical modelling

The association of prestroke dependency with the main outcomes was estimated by calculating odds ratios (ORs) with 95% CI, using multivariate binary logistic regression models. The models were adjusted for a priori defined, well-known confounders described in the literature. The models for poor outcome and death at 3 months were adjusted for age,^35^ NIHSS at admission,^35^^,^^36^ sex,^37^ EVT complications,^36^ unsuccessful recanalisation (mTICI 0–2a),^35^^,^^36^ bridging therapy,^38^ admission glucose,^39^ prior atrial fibrillation,^40^ prior ischaemic stroke^36^ and time from symptom onset or last seen well to groin^41^ (10 covariates total). The models for sICH and aICH were adjusted for age,^42^ sex,^43^ NIHSS at admission,^36^^,^^42^ EVT complications,^36^ bridging therapy,^36^ admission systolic blood pressure^42^ and time from symptom onset or last seen well to groin^36^^,^^42^^,^^43^ (7 covariates total). The model for unsuccessful recanalisation was adjusted for age,^44–46^ sex,^44^^,^^46^ NIHSS at admission,^44^^,^^46^ use of general anaesthesia during EVT,^45^ bridging therapy,^44^^,^^46^ admission systolic blood pressure,^44^^,^^46^ prior atrial fibrillation,^44^^,^^46^ prior diabetes mellitus^44^^,^^46^ and time from symptom onset or last seen well to groin^44^^,^^46^ (9 covariates total).

The main analysis is the full models using the imputed dataset. As an additional analysis, we added an interaction term of unsuccessful recanalisation and prestroke dependency for the main analysis of poor outcome, to study whether there was a differential effect on the outcome between the 2 study groups according to recanalisation status.

The following sensitivity analyses were performed: (i) The same models were also calculated using only the complete cases to assess the bias caused by the missing data and their imputation. (ii) Using the imputed data sets, simpler models using only NIHSS at admission and age were calculated for all outcomes to assess the potential gain from using more complex models. (iii) Propensity score matching using the imputed datasets as an alternative approach to covariate adjustment. Detailed methods and covariate balance assessments for the propensity score matching analysis are provided in the Supplementary Methods.

### Independent data access and analysis

Ines Piot, Lukas S. Enz, Stefan T. Engelter and Henrik Gensicke had full access to all the data in the study and take responsibility for its integrity and the data analysis.

### Ethics statement

Study approval was given by the ethics committee in Basel, Switzerland. Each participating centre also obtained local ethical approval in its country.

## Results

**Figure 1:**
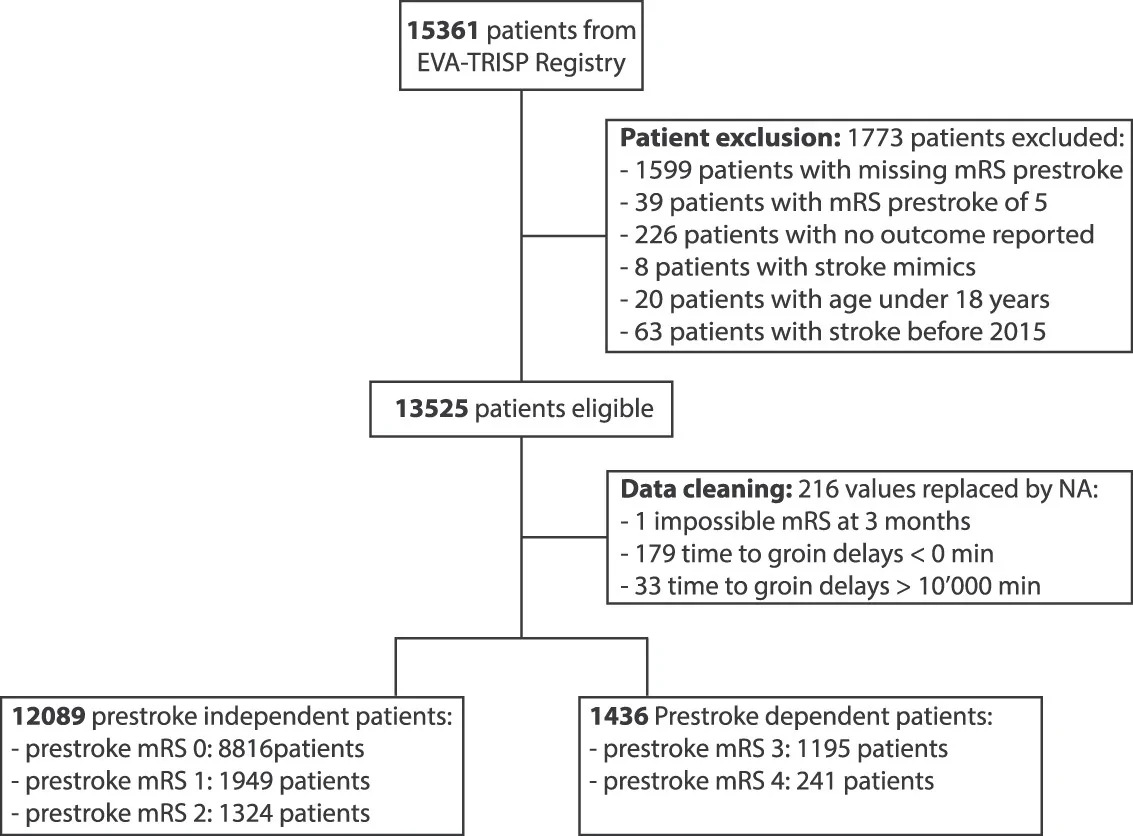
Inclusion of patients from EVA-TRISP registry and definition of main study groups prestroke-independent (mRS 0–2) and prestroke-dependent (mRS 3–4). Abbreviations: EVA-TRISP = EndoVascular Therapy and ThRombolysis in Ischemic Stroke Patients; NA = not available.

Of a total of 15,361 patients, data from 13,525 patients were eligible for analysis (1599 excluded due to missing prestroke mRS, 39 patients due to prestroke mRS 5, 226 due to none of the outcomes being reported, 8 patients due to being stroke mimics, 20 patients aged under 18, 63 due to stroke onset before the year 2015) (Figure 1; Table S2).

**Figure 2:**
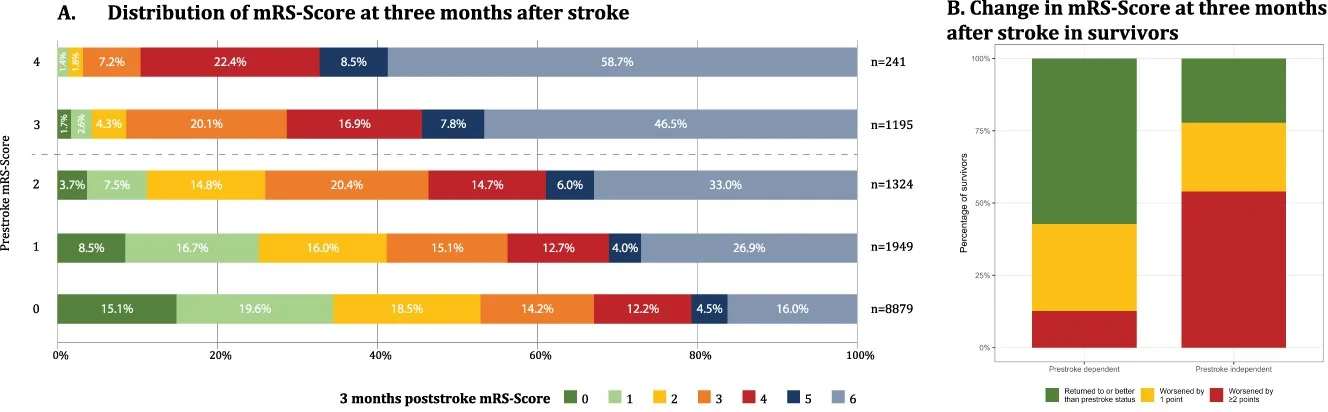
(A) Modified Rankin scale distribution at baseline and after 3 months according to prestroke mRS. (B) Distribution of change in mRS from prestroke baseline among surviving prestroke-independent and prestroke-dependent patients.

From the eligible patients, 1436 (10.6%) were prestroke-dependent (mRS 3–4, study group) and 12,089 (89.4%) were prestroke-independent (mRS 0–2, control group) (Figure 1). Of the prestroke-dependent patients, 1195 (83.2%) had a prestroke mRS score of 3 and 241 (16.8%) had a prestroke mRS score of 4. Of the prestroke-independent patients, 8816 (72.9%) had a prestroke mRS score of 0, 1949 (16.1%) had a prestroke mRS score of 1 and 1324 (11.0%) had a prestroke mRS score of 2 (Figure 1). The distribution of mRS at 3 months for each prestroke mRS score is shown in Figure 2A and the distribution of the delta mRS in survivors (prestroke mRS vs mRS at 3 months) is shown in Figure 2B. During data cleaning, 1 impossible mRS at 3 months, 179 negative time to groin delays and 33 time to groin delays > 10,000 min were replaced with a missing value due to being impossible or highly implausible (Figure 1).

### Baseline characteristics

Prestroke-dependent patients were older (median 84 vs 73 years), more often women (61.0% vs 46.7%), had higher NIHSS scores on admission (median 16 vs 14), were less likely to receive IVT (34.5% vs 48.6%) and more likely to receive general anaesthesia (71.4% vs 63.5%) (all adj. *P* < .01; Table 1). The number of passes during EVT was only reported from 6 centres, but there was no evidence of a difference between the 2 groups. Early termination of EVT was numerically more common in prestroke-dependent patients (14.7% vs 11.6%, adj. *P*-value .13; Table 1). The stroke territories and aetiologies were similar in both groups except for a higher rate of cardioembolic aetiology in the prestroke-dependent group (52.2% vs 42.7%). Prestroke-dependent patients more often had cerebro- and cardiovascular risk factors (previous stroke, coronary artery disease, atrial fibrillation, diabetes mellitus and hypertension) except for current smoking, which was more pronounced in the previously independent group (12.2% vs 21.3%). Hypercholesterolemia was similarly distributed in both groups (51.0% vs 49.6%) (Table 1).

### Unadjusted outcomes

The frequency of poor functional outcome in stroke survivors (42.8% vs 40.0%; OR_unadjusted_ 1.12; 95% CI, 0.96–1.31) did not differ significantly between the groups. Prestroke-dependent patients died more often during follow-up (48.6% vs 19.6%; OR_unadjusted_ 3.88; 95% CI, 3.45–4.36). Symptomatic ICH (3.8% vs 4.6%; OR_unadjusted_ 0.81; 95% CI, 0.60–1.08) and aICH (25.8% vs 26.9%; OR_unadjusted_ 0.95; 95% CI, 0.83–1.08) did not differ significantly between the groups. Prestroke-dependent patients more often had unsuccessful recanalisation (31.0% vs 26.6%; OR_unadjusted_ 1.24; 95% CI, 1.09–1.40).

### Missing data and multiple imputation

Over all data given in Table 1, 10.8% of data were missing (ie, 89.2% data completeness). This was mainly caused by items not reported by all centres (stroke territory, ASPECT score, EVT complications, intra-arterial thrombolysis, EVT number of passes, EVT stopped early, coronary artery disease, TOAST categories and status of recanalisation). Without these items, only 3.4% of the data were missing (ie, 96.6% data completeness). The main source of missing data was non-reporting by specific centres, and we therefore assumed it to be missing at random.

### Multivariate logistic regression models

**Figure 3:**
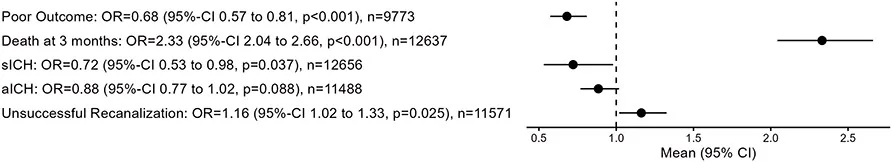
Effect of prestroke dependency on outcomes. Odds ratios larger than 1 denote larger risk for the previously dependent patients. Results are from the full models using the imputed data set. Abbreviation: aICH: any intracranial haemorrhage.

There were decreased odds of poor outcome in survivors in the previously dependent group (OR_adjusted_ = 0.68 [95% CI, 0.57–0.81; *P* < .001], *n* = 9773; Figure 3), while the odds of death were increased (OR_adjusted_ = 2.33 [95% CI 2.04–2.66; *P* < .001], *n* = 12,637). The odds of sICH were decreased in the previously dependent group (OR_adjusted_ = 0.72 [95% CI, 0.53–0.98; *P* = .037], *n* = 12,656), while the odds of aICH did not show evidence of a difference (OR_adjusted_ = 0.88 [95% CI, 0.77–1.02; *P* = .088], *n* = 11,488). Furthermore, there were increased odds of unsuccessful recanalisation (OR_adjusted_ = 1.16 [95% CI, 1.02–1.33; *P* = .025], *n* = 11,571) in the previously dependent patients. Full model results showing all variables are given in Table S3.

We further investigated whether there might be a differential effect on poor outcome in survivors of the recanalisation status given prestroke dependency by adding an interaction term to the model. There was no evidence for this (OR_adjusted_ = 0.80 [95% CI, 0.54–1.20; *P* = .278], *n* = 9773), indicating that the recanalisation status is of similar importance in both groups concerning poor outcome.

### Sensitivity analyses

The following sensitivity analyses were performed: (1) complete case analysis, (2) simpler models using only age and NIHSS on admission as covariates and using the imputed datasets, (3) propensity score matching (for details see Supplementary Methods and Figures S1 and S2). All 3 sensitivity analyses supported the evidence from the main analysis above for poor outcome in survivors, death, aICH and unsuccessful recanalisation.

Notably, the odds of poor outcome were reduced in all models for prestroke-dependent patients. This stands in contrast to the nearly identical raw risk of poor outcome (Table 1; 42.8% vs 40.0%). As suggested by the simpler model, these results are mainly driven by the increased age and NIHSS at admission of the prestroke-dependent patients.

Furthermore, despite similar point estimates of the odds ratios for sICH (range from 0.72 to 0.79 across all 4 models), only the main analysis reached a *P*-value of .037, while the 3 sensitivity analyses showed *P*-values from .061 to .160, providing less evidence for lower odds of sICH in the prestroke-dependent group.

### Putative contributors to prestroke dependency

A subset of centres retrospectively reported on possible contributing conditions to prestroke dependency (Amsterdam, Basel, Belgrade, Bern, Bologna, Brescia, Lausanne, Lisbon, Zurich). For this, medical records of prestroke-dependent patients were scanned for predefined categories of diseases (Table 2). The most common reported categories were internal illnesses (69.6% of all prestroke-dependent patients), traumatic or degenerative bone diseases (58.0%) and neurological diseases (dementia 33.1%, prior ischaemic stroke 26.9%, other neurological diseases 28.6%).

**Table 2:** Putative contributors to prestroke dependency.

Variable	Number of patients from prestroke-dependent group (total *n* = 1436)
**Internal disease**	457/657 (69.6%)
**Traumatic or degenerative bone disease**	347/598 (58%)
**Dementia**	224/677 (33.1%)
**Other neurological disease**	192/672 (28.6%)
**Prior ischaemic stroke**	357/1327 (26.9%)
**Active oncological disease**	125/680 (18.4%)
**Psychiatric disease**	125/676 (18.5%)
**Parkinson’s disease**	29/561 (5.2%)
**Epilepsy**	23/559 (4.1%)

Complete information across all predefined categories was available for 543 of 1436 prestroke-dependent patients (37.8%). Within this subset, substantial overlap between categories was observed: the majority of patients (400, 73.7%) met criteria for 2 or more categories, whereas 115 (21.2%) fit exactly 1 category and only 28 patients (5.2%) did not meet any predefined category. These findings indicate that prestroke dependency is typically multifactorial, with frequent overlap between contributing conditions rather than a single isolated cause.

## Discussion

The key results of our study are: (1) among stroke survivors, prestroke dependency was not associated with worse functional outcomes after adjustment for baseline differences and there was no evidence of a differential effect of the recanalisation status given prestroke dependency, indicating that successful recanalisation is equally important for a good outcome in both groups, (2) prestroke-dependent patients had higher odds of 90-day mortality, (3) there was no evidence of an increased risk of sICH and aICH in the prestroke-dependent patients and (4) prestroke dependency was associated with higher odds of unsuccessful recanalisation.

Functional outcomes among survivors showed similar to slightly decreased odds of poor functional outcome in prestroke-dependent patients, a pattern that has previously been reported in patients treated with IVT and in multicentre EVT studies.^14^^,^^27^^,^^47^ Adjusted estimates tended to favour dependent patients, while unadjusted odds were similar between groups. This finding should be interpreted cautiously and may reflect 3 sources of bias, making outcomes inherently different across groups: (1) prestroke-dependent patients were likely more rigorously selected for EVT, (2) the mRS is an ordinal and non-linear scale, and deterioration is not equally distributed across baseline levels and (3) management of comorbid conditions during acute care and rehabilitation may have improved functional outcomes. Regarding the second point, worsening from mRS 4 to 5 is not necessarily comparable to worsening from mRS 0 to 3, and our outcome definition may favour patients with higher baseline mRS scores. Mortality was more than twice as high in prestroke-dependent patients. This finding is consistent with prior studies, which have almost uniformly reported higher mortality rates in prestroke-dependent populations, often of similar magnitude to those observed in the present analysis.^9^^,^^10^^,^^13^^,^^17^

Safety outcomes were comparable with previous reports,^9^^,^^10^^,^^13^^,^^14^ and we hypothesise that the tendency towards lower odds of sICH in our cohort may have been caused by a more rigorous patient selection in the prestroke-dependent group, biasing the outcomes. Furthermore, as the prestroke-dependent patients presented with a higher NIHSS score on average but the definition of sICH requires an increase of at least 4 points on the NIHSS, we hypothesise that sICH may be relatively underreported in prestroke-dependent patients due to a ceiling effect, where comparable haemorrhages may not meet the sICH criteria due to an already overall higher NIHSS score. Our analysis of aICH, however, did not reveal any evidence of an increased risk in haemorrhages in the prestroke-dependent patients.

With respect to procedural efficacy, our data suggest a modestly higher likelihood of unsuccessful recanalisation in prestroke-dependent patients, with an absolute difference of 4.2%. The magnitude of difference observed in our cohort aligns with other reports, such as the approximately 5% difference described by Larsson et al.,^13^ which did not reach statistical significance in smaller samples.

There was no evidence of fewer passes during EVT or a higher rate of early stopping of EVT in prestroke-dependent patients, though early stopping was numerically modestly more common. We cannot exclude that a lower threshold for discontinuing procedures in frail patients with substantial premorbid disability contributed to this finding, but given the modest absolute difference and lack of statistical significance, this remains speculative. Detailed data on operator decision-making and rescue strategies were not available to us for further investigation.

Our findings align with previous observational data regarding the characteristics of the prestroke-dependent cohort. These patients were more often female, older, had higher NIHSS scores and carried a greater cardiovascular burden—factors that are known to be associated with higher pre- and poststroke dependency and mortality.^13^^,^^17^^,^^48–50^ The leading causes of stroke in both prestroke-dependent and -independent patients were cardioembolism and large artery atherosclerosis.

The main reported contributors of prestroke dependency were internal illnesses, traumatic or degenerative bone disease, dementia and prior ischaemic stroke. Previous studies have similarly identified multimorbidity, prior stroke, cardiopulmonary disease and cognitive impairment as common correlates of prestroke dependency.^27^^,^^51^ Due to incomplete reporting and the absence of systematic classification of causative pathologies, we were unable to further analyse these associations, and the observed relationships should therefore be considered associative rather than causal. More detailed cohort studies are needed to clarify potential links between specific causes of prestroke dependency and post-EVT outcomes.

Several analyses addressing closely related questions from observational studies, along with 2 recent multicentre investigations, provide important context for our findings. One overarching question is the comparison of EVT vs best medical therapy (BMT) in prestroke-dependent patients. Overall, studies have reported better functional outcomes with EVT compared with BMT,^47^^,^^52^ even in late-window EVT,^53^ while one very recent study interestingly reported unchanged odds of transitioning to comfort care.^54^ This observation highlights the complexity of clinical trajectories in prestroke-dependent patients, which may not be fully captured by the mRS alone.

The 2 largest registry-based analyses addressing this question used similar definitions of prestroke dependency. The 2024 Czech national registry study by Ganesh et al.^15^ included 159 EVT-treated patients with prestroke mRS > 2 for the outcome analyses, whereas the 2025 Italian registry study by Naldi et al.^16^ included 429 patients with prestroke mRS 3–4 for the outcome analyses. It is notable that the Czech study defined its primary outcome using ΔmRS, while the Italian study used no change from prestroke mRS at 3 months, an approach that may modestly favour good outcomes in prestroke-dependent patients, as larger mRS increments are harder to achieve at higher baseline scores. In the Czech study, prestroke-dependent patients experienced slower treatment times and overall worse outcomes compared with independent patients. Higher mortality and institutionalisation rates were consistent with prior reports, while functional outcomes appeared slightly more pessimistic, possibly reflecting cohort-specific or procedural factors. In contrast, the Italian study, which stratified outcomes by age and baseline mRS, reported trends similar to ours, including comparable rates of return to prestroke functional status but higher mortality and lower recanalisation rates among dependent patients.

The present study complements these 2 studies by including the largest international cohort of prestroke-dependent patients (total 1474) undergoing EVT to date. Missing outcome data were minimal and addressed using multiple imputation. Importantly, the inclusion of a substantial proportion of patients with prestroke mRS 4–5 (total 279) enabled analyses in individuals with more severe baseline disability, a group that remains underrepresented in prior studies. The multinational design and characterisation of prestroke-dependency aetiologies further address existing evidence gaps. Furthermore, our study spans almost a decade of EVT practice (2015-2023), during which endovascular devices and treatment workflows evolved substantially. While this may introduce temporal heterogeneity, it also enhances the generalisability of our findings by reflecting real-world outcomes across different phases of EVT implementation and maturation.

Several limitations should be acknowledged, foremost a potential selection bias of the study population. Because only EVT-treated patients were included, the study population reflects local treatment decisions rather than the full spectrum of prestroke-dependent patients presenting with large vessel occlusion. Clinicians may have preferentially selected patients with greater recovery potential or fewer comorbidities, while excluding those considered unlikely to benefit. As a result, the findings should be interpreted as applying to selected prestroke-dependent patients who underwent EVT rather than to all prestroke-dependent stroke patients. Furthermore, the mRS has inherent limitations, including uneven intervals and differential capture of physical vs cognitive disability. We used a dichotomised definition of poor outcome for simplicity, patient-centred relevance and consistency with prior studies, while recognising that higher mRS scores reflect larger increments of disability and that mRS 3–4 represents substantial impairment. Outcome definitions in this population remain debated, and alternative approaches such as ΔmRS have been proposed. Furthermore, we did not assess the type or intensity of rehabilitation, which may have influenced outcomes. Another limitation is that data collection and revision are performed locally without central adjudication or external auditing. Consequently, inter-centre variability in data collection and interpretation cannot be excluded. However, the multicentre design also reflects real-world clinical practice and may enhance the generalisability of the findings. As a non-randomised retrospective registry study, residual confounding cannot be excluded.

## Conclusion

In conclusion, our study is the first to combine an international design with high data completeness and large-scale analyses in patients with prestroke dependency undergoing EVT. It provides further evidence that selected acute ischaemic stroke patients with prestroke dependency can achieve meaningful outcomes after EVT and should not be routinely excluded from consideration for treatment solely on the basis of baseline disability. Our findings should not be interpreted as supporting EVT in all patients with prestroke dependency; rather, they suggest that baseline disability should be considered alongside other established prognostic factors when making individualised treatment decisions. While these patients face a higher risk of mortality, survivors can achieve or preserve meaningful functional status. Collectively, our findings challenge practices that withhold EVT solely on the basis of baseline disability and underscore the need for updated clinical guidelines and randomised trials in this population.

## Supplementary Material

Supplementary_material_aakag099

## Data Availability

Datasets generated or analysed within the present study are available from the corresponding author upon reasonable request. In each such case, compliance of data sharing with individual processes of patient consenting in participating centres is required. Final decision on data sharing will be made by consensus of the EVA-TRISP collaborators.

## References

[1] Powers WJ, Rabinstein AA, Ackerson T, et al. Guidelines for the early management of patients with acute ischemic stroke: 2019 update to the 2018 guidelines for the early management of acute ischemic stroke: a guideline for healthcare professionals from the American Heart Association/American Stroke Association. Stroke. 2019;50:e344–e418. 10.1161/STR.0000000000000211 31662037

[2] Turc G, Bhogal P, Fischer U, et al. European Stroke Organisation (ESO)—European Society for Minimally Invasive Neurological Therapy (ESMINT) guidelines on mechanical thrombectomy in acute ischemic stroke. J Neurointerv Surg. 2023;15:e8. 10.1136/neurintsurg-2018-014569 30808653

[3] Ganesh A, Luengo-Fernandez R, Pendlebury ST, Rothwell PM, on behalf of the Oxford Vascular Study. Long-term consequences of worsened poststroke status in patients with premorbid disability. Stroke. 2018;49:2430–2436. 10.1161/strokeaha.118.022416 30355105 PMC6159688

[4] Krahn GL, Walker DK, Correa-De-Araujo R. Persons with disabilities as an unrecognized health disparity population. Am J Public Health. 2015;105 Suppl 2:S198–S206. 10.2105/ajph.2014.302182 25689212 PMC4355692

[5] Jovin TG, Chamorro A, Cobo E, et al. Thrombectomy within 8 hours after symptom onset in ischemic stroke. N Engl J Med. 2015;372:2296–2306. 10.1056/NEJMoa1503780 25882510

[6] Saver JL, Goyal M, Bonafe A, et al. Stent-retriever thrombectomy after intravenous t-PA vs. t-PA alone in stroke. N Engl J Med. 2015;372:2285–2295. 10.1056/NEJMoa1415061 25882376

[7] Fransen PS, Beumer D, Berkhemer OA, et al. MR CLEAN, a multicenter randomized clinical trial of endovascular treatment for acute ischemic stroke in the Netherlands: study protocol for a randomized controlled trial. Trials. 2014;15:343. 10.1186/1745-6215-15-343 25179366 PMC4162915

[8] Ospel JM, Singh N, Almekhlafi MA, et al. Early recanalization with alteplase in stroke because of large vessel occlusion in the ESCAPE trial. Stroke. 2021;52:304–307. 10.1161/strokeaha.120.031591 33213288

[9] Goldhoorn RB, Verhagen M, Dippel DWJ, et al. Safety and outcome of endovascular treatment in prestroke-dependent patients. Stroke. 2018;49:2406–2414. 10.1161/strokeaha.118.022352 30355090

[10] Oesch L, Arnold M, Bernasconi C, et al. Impact of pre-stroke dependency on outcome after endovascular therapy in acute ischemic stroke. J Neurol. 2021;268:541–548. 10.1007/s00415-020-10172-3 32865630 PMC7880932

[11] Regenhardt RW, Young MJ, Etherton MR, et al. Toward a more inclusive paradigm: thrombectomy for stroke patients with pre-existing disabilities. J Neurointerv Surg. 2021;13:865–868. 10.1136/neurintsurg-2020-016783 33127734 PMC8365380

[12] Millán M, Ramos-Pachón A, Dorado L, et al. Predictors of functional outcome after thrombectomy in patients with prestroke disability in clinical practice. Stroke. 2022;53:845–854. 10.1161/strokeaha.121.034960 34702065

[13] Larsson A, Karlsson C, Rentzos A, et al. Do patients with large vessel occlusion ischemic stroke harboring prestroke disability benefit from thrombectomy? J Neurol. 2020;267:2667–2674. 10.1007/s00415-020-09882-5 32410019 PMC7419353

[14] Ducroux C, Derex L, Nourredine M, et al. Successful thrombectomy is beneficial in patients with pre-stroke disability: results from an international multicenter cohort study. J Neuroradiol. 2023;50:59–64. 10.1016/j.neurad.2022.03.006 35341899

[15] Ganesh A, Volny O, Kovacova I, et al. Utilization, workflow, and outcomes of endovascular thrombectomy in patients with vs without premorbid disability in a national registry. Neurol Clin Pract. 2024;14:e200341. 10.1212/cpj.0000000000200341 39185095 PMC11341008

[16] Naldi A, D'Agata F, Pracucci G, et al. Mechanical thrombectomy in prestroke disability: data from the Italian Endovascular Stroke Registry. Stroke. 2025;56:850–857. 10.1161/strokeaha.124.048997 40052284 PMC11932441

[17] Leker RR, Gavriliuc P, Yaghmour NE, Gomori JM, Cohen JE. Increased risk for unfavorable outcome in patients with pre-existing disability undergoing endovascular therapy. J Stroke Cerebrovasc Dis. 2018;27:92–96. 10.1016/j.jstrokecerebrovasdis.2017.08.007 28882658

[18] Adamou A, Gkana A, Mavrovounis G, Beltsios ET, Kastrup A, Papanagiotou P. Outcome of endovascular thrombectomy in pre-stroke dependent patients with acute ischemic stroke: a systematic review and meta-analysis. Front Neurol. 2022;13:880046. 10.3389/fneur.2022.880046 35572918 PMC9097509

[19] Yang JC, Bao QJ, Guo Y, et al. Endovascular thrombectomy in acute ischemic stroke patients with prestroke disability (mRS ≥ 2): a systematic review and meta-analysis. Front Neurol. 2022;13:971399. 10.3389/fneur.2022.971399 36188370 PMC9520304

[20] Bala F, Beland B, Mistry E, Almekhlafi MA, Goyal M, Ganesh A. Endovascular treatment of acute ischemic stroke in patients with pre-morbid disability: a meta-analysis. J Neurointerv Surg. 2023;15:343–349. 10.1136/neurintsurg-2021-018573 35292569

[21] Zhao H, Bai X, Li W, et al. Influence of pre-stroke dependency on safety and efficacy of endovascular therapy: a systematic review and meta-analysis. Front Neurol. 2022;13:956958. 10.3389/fneur.2022.956958 36212663 PMC9532553

[22] Nordanstig A, Curtze S, Gensicke H, et al. EndoVAscular treatment and ThRombolysis for Ischemic Stroke Patients (EVA-TRISP) registry: basis and methodology of a pan-European prospective ischaemic stroke revascularisation treatment registry. BMJ Open. 2021;11:e042211. 10.1136/bmjopen-2020-042211 PMC8354282 34373287

[23] Scheitz JF, Gensicke H, Zinkstok SM, et al. Cohort profile: Thrombolysis in Ischemic Stroke Patients (TRISP): a multicentre research collaboration. BMJ Open. 2018;8:e023265. 10.1136/bmjopen-2018-023265 PMC6150152 30224398

[24] Traenka C, Lorscheider J, Hametner C, et al. Recanalization therapies for large vessel occlusion due to cervical artery dissection: a cohort study of the EVA-TRISP collaboration. J Stroke. 2023;25:272–281. 10.5853/jos.2022.03370 37282374 PMC10250869

[25] Barber PA, Demchuk AM, Zhang J, Buchan AM. Validity and reliability of a quantitative computed tomography score in predicting outcome of hyperacute stroke before thrombolytic therapy. ASPECTS Study Group. Alberta Stroke Programme Early CT Score. Lancet. 2000;355:1670–1674. 10.1016/s0140-6736(00)02237-6 10905241

[26] Adams HP Jr, Bendixen BH, Kappelle LJ, et al. Classification of subtype of acute ischemic stroke. Definitions for use in a multicenter clinical trial. TOAST. Trial of Org 10172 in Acute Stroke Treatment. Stroke. 1993;24:35–41. 10.1161/01.str.24.1.35 7678184

[27] Gensicke H, Strbian D, Zinkstok SM, et al. Intravenous thrombolysis in patients dependent on the daily help of others before stroke. Stroke. 2016;47:450–456. 10.1161/strokeaha.115.011674 26797662

[28] Karlinski M, Kobayashi A, Czlonkowska A, et al. Role of preexisting disability in patients treated with intravenous thrombolysis for ischemic stroke. Stroke. 2014;45:770–775. 10.1161/strokeaha.113.003744 24496395

[29] Hacke W, Kaste M, Bluhmki E, et al. Thrombolysis with alteplase 3 to 4.5 hours after acute ischemic stroke. N Engl J Med. 2008;359:1317–1329. 10.1056/NEJMoa0804656 18815396

[30] Hacke W, Kaste M, Fieschi C, et al. Randomised double-blind placebo-controlled trial of thrombolytic therapy with intravenous alteplase in acute ischaemic stroke (ECASS II). Second European-Australasian Acute Stroke Study Investigators. Lancet. 1998;352:1245–1251. 10.1016/s0140-6736(98)08020-9 9788453

[31] Zaidat OO, Yoo AJ, Khatri P, et al. Recommendations on angiographic revascularization grading standards for acute ischemic stroke: a consensus statement. Stroke. 2013;44:2650–2663. 10.1161/strokeaha.113.001972 23920012 PMC4160883

[32] Banks JL, Marotta CA. Outcomes validity and reliability of the modified Rankin scale: implications for stroke clinical trials: a literature review and synthesis. Stroke. 2007;38:1091–1096. 10.1161/01.STR.0000258355.23810.c6 17272767

[33] von Hippel PT . Regression with missing Ys: an improved strategy for analyzing multiply imputed data. Sociol Methodol. 2007;37:83–117. 10.1111/j.1467-9531.2007.00180.x

[34] Rubin DB . Multiple Imputation for Nonresponse in Surveys. John Wiley and Sons; 1987.

[35] Rogalewski A, Klein N, Friedrich A, et al. Functional long-term outcome following endovascular thrombectomy in patients with acute ischemic stroke. Neurol Res Pract. 2024;6:2. 10.1186/s42466-023-00301-4 38297374 PMC10832147

[36] Ironside N, Chen CJ, Chalhoub RM, et al. Risk factors and predictors of intracranial hemorrhage after mechanical thrombectomy in acute ischemic stroke: insights from the Stroke Thrombectomy and Aneurysm Registry (STAR). J Neurointerv Surg. 2023;15:e312–e322. 10.1136/jnis-2022-019513 36725360 PMC10962911

[37] Demel SL, Reeves M, Xu H, et al. Sex differences in endovascular therapy for ischemic stroke: results from the get with the guidelines-stroke registry. Stroke. 2022;53:3099–3106. 10.1161/strokeaha.122.038491 35880521

[38] Qin B, Wei T, Gao W, et al. Real-world setting comparison of bridging therapy versus direct mechanical thrombectomy for acute ischemic stroke: a meta-analysis. Clinics (Sao Paulo). 2024;79:100394. 10.1016/j.clinsp.2024.100394 38820696 PMC11177057

[39] Lau LH, Lew J, Borschmann K, Thijs V, Ekinci EI. Prevalence of diabetes and its effects on stroke outcomes: a meta-analysis and literature review. J Diabetes Investig. 2019;10:780–792. 10.1111/jdi.12932 PMC6497593 30220102

[40] Kobeissi H, Ghozy S, Seymour T, et al. Outcomes of patients with atrial fibrillation following thrombectomy for stroke: a systematic review and meta-analysis. JAMA Netw Open. 2023;6:e2249993. 10.1001/jamanetworkopen.2022.49993 36607633 PMC9857225

[41] Fransen PS, Berkhemer OA, Lingsma HF, et al. Time to reperfusion and treatment effect for acute ischemic stroke: a randomized clinical trial. JAMA Neurol. 2016;73:190–196. 10.1001/jamaneurol.2015.3886 26716735

[42] Dong S, Yu C, Wu Q, et al. Predictors of symptomatic intracranial hemorrhage after endovascular thrombectomy in acute ischemic stroke: a systematic review and meta-analysis. Cerebrovasc Dis. 2023;52:363–375. 10.1159/000527193 36423584

[43] Hao Z, Yang C, Xiang L, Wu B, Liu M. Risk factors for intracranial hemorrhage after mechanical thrombectomy: a systematic review and meta-analysis. Expert Rev Neurother. 2019;19:927–935. 10.1080/14737175.2019.1632191 31200607

[44] Deng G, Xiao J, Yu H, et al. Predictors of futile recanalization after endovascular treatment in acute ischemic stroke: a meta-analysis. J Neurointerv Surg. 2022;14:881–885. 10.1136/neurintsurg-2021-017963 34544824

[45] Kniep H, Meyer L, Broocks G, et al. Thrombectomy for M2 occlusions: predictors of successful and futile recanalization. Stroke. 2023;54:2002–2012. 10.1161/strokeaha.123.043285 37439204

[46] Shen H, Killingsworth MC, Bhaskar SMM. Comprehensive meta-analysis of futile recanalization in acute ischemic stroke patients undergoing endovascular thrombectomy: prevalence, factors, and clinical outcomes. Life (Basel). 2023;13:13. 10.3390/life13101965 PMC10608522 37895347

[47] Siegler JE, Qureshi MM, Nogueira RG, et al. Endovascular vs medical management for late anterior large vessel occlusion with prestroke disability: analysis of CLEAR and RESCUE-Japan. Neurology. 2023;100:e751–e763. 10.1212/wnl.0000000000201543 36332983 PMC9969918

[48] Quinn TJ, Taylor-Rowan M, Coyte A, et al. Pre-stroke modified Rankin scale: evaluation of validity, prognostic accuracy, and association with treatment. Front Neurol. 2017;8:275. 10.3389/fneur.2017.00275 28659859 PMC5468801

[49] Petrea RE, Beiser AS, Seshadri S, Kelly-Hayes M, Kase CS, Wolf PA. Gender differences in stroke incidence and poststroke disability in the Framingham heart study. Stroke. 2009;40:1032–1037. 10.1161/strokeaha.108.542894 19211484 PMC2676725

[50] Leker RR, Cohen JE, Horev A, et al. Impact of previous stroke on outcome after thrombectomy in patients with large vessel occlusion. Int J Stroke. 2019;14:887–892. 10.1177/1747493019841244 30947643

[51] Benali F, Kappelhof M, Ospel J, et al. Benefit of successful reperfusion achieved by endovascular thrombectomy for patients with ischemic stroke and moderate pre-stroke disability (mRS 3): results from the MR CLEAN Registry. J Neurointerv Surg. 2023;15:433–438. 10.1136/neurintsurg-2022-018853 35414601

[52] Tanaka K, Yamagami H, Yoshimoto T, et al. Endovascular therapy for acute ischemic stroke in patients with prestroke disability. J Am Heart Assoc. 2021;10:e020783. 10.1161/jaha.121.020783 34284599 PMC8475666

[53] Maestrini I, Rocchi L, Diana F, et al. Outcomes and safety of endovascular treatment from 6 to 24 hours in patients with a pre-stroke moderate disability (mRS 3): a multicenter retrospective study. J Neurointerv Surg. 2024;17:546–552. 10.1136/jnis-2024-021634 38811146

[54] Awad A, Young MJ, Andreev A, et al. Endovascular thrombectomy for large vessel occlusion stroke patients with baseline disability: long-term outcomes and end-of-life care. Clin Neurol Neurosurg. 2025;249:108768. 10.1016/j.clineuro.2025.108768 39923494

