# Supplementary material for: Endovascular therapy in acute ischaemic stroke patients with prestroke dependency—an EVA-TRISP cohort study
Source: Eur Stroke J. 2026 Aug 21;11(8):aakag099. doi: 10.1093/esj/aakag099 (PMC13496238; doi:10.1093/esj/aakag099)
Supplement: Supplementary file 1 [file supplementary_material_aakag099.zip › Supplement_EVATRISP_PreviouslyDependent_2026_Apr_28_ESJ_Revision1_aakag099.docx]

**Supplements**

**Supplementary Methods**

*Details of propensity score matching*

Using the imputed data sets, propensity score matching was performed to reduce the potential influence of between-group differences in key baseline variables on the outcome analyses. We matched patients according to their propensity to be in the previously dependent or independent group. The propensity score was based on the complex models above and patients were matched in a 3:1 ratio with nearest-neighbor matching within a caliper of 0.05 standard deviations of the propensity score. A higher ratio did not further increase the number of cases used. Propensity scores weighted modelling approaches were not possible due to lacking similarity between the groups. We report the unadjusted and adjusted covariate balancing. We then performed unadjusted logistic regression analyses in the matched dataset to assess differences in the outcomes.

Matching was successful and more than 98.7% of the previously dependent patients could be matched (i.e. 1.3% of patients in the previously dependent group were discarded for this analysis). Distribution of the unadjusted and adjusted covariates are given in the supplements (**Supplementary Figure 2**).

**Supplementary Table 1.** Details of patient variables.

| **Variable Name** | **Variable Type** |
| --- | --- |
| Prestroke mRS | Integer, range 0 to 5 |
| Age [Years] | Integer |
| Sex | Binary (M/F) |
| NIHSS Admission | Integer, range 0 to 42 |
| Stroke territory | Ordinal (Anterior, Posterior, Both) |
| ASPECTS | Integer, range 10 to 0 |
| Time to Groin | Integer |
| Bridging therapy | Binary (Yes/No) |
| EVT Complications | Binary (Yes/No) |
| EVT Intraarterial thrombolysis | Binary (Yes/No) |
| EVT Mechanical thrombectomy | Binary (Yes/No) |
| EVT General anesthesia | Binary (Yes/No) |
| EVT Number of passes | Integer |
| EVT Stopped early | Binary (Yes/No) |
| Admission Systolic Blood pressure [mmHg] | Integer |
| Admission Glucose [mmol/l] | Numeric |
| Prior ischemic stroke | Binary (Yes/No) |
| Coronary artery disease | Binary (Yes/No) |
| Atrial fibrillation | Binary (Yes/No) |
| Diabetes mellitus | Binary (Yes/No) |
| Arterial hypertension | Binary (Yes/No) |
| Hypercholesterolemia | Binary (Yes/No) |
| Current smoking or stopped < 2 years | Binary (Yes/No) |
| Prior platelet inhibition | Binary (Yes/No) |
| Prior oral anticoagulation | Binary (Yes/No) |
| TOAST classification | Ordinal (Large artery atherosclerosis, cardioembolic stroke, stroke of other determined cause, more than one, stroke of undetermined cause) |
| Poor outcome at 3 months in survivors | Binary (Yes/No) |
| Death at 3 months | Binary (Yes/No) |
| Symptomatic intracranial hemorrhage | Binary (Yes/No) |
| Any intracranial hemorrhage | Binary (Yes/No) |
| Unsuccessful Recanalization | Binary (Yes/No) |

**Supplementary Table 2.** Centres and time of data acquisition:

| **Centres (city, country)** | **Time table** | **Number of Patients** |
| --- | --- | --- |
| Amsterdam, Netherlands | 01/16-03/22 | 578 |
| Basel, Switzerland | 01/15-07/22 | 814 |
| Belgrad, Serbia | 01/18-12/21 | 239 |
| Berlin, Germany | 2016-2020 | 842 |
| Bern, Switzerland | 02/15-10/22 | 2’272 |
| Bologna, Italy | 09/18-09/22 | 698 |
| Brescia, Italy | 01/15-07/21 | 503 |
| Göteborg, Sweden | 01/15-12/20 | 1’369 |
| Helsinki, Finland | 01/16-12/20 | 935 |
| Jerusalem, Israel | 01/15-03/22 | 553 |
| Lausanne, Switzerland | 2015-2022 | 1’136 |
| Lisbon, Portugal | 01/16-04/22 | 581 |
| Lugano, Switzerland | 02/15-01/23 | 501 |
| St. Gallen, Switzerland | 01/15-01/22 | 838 |
| Tel-Aviv, Israel | 06/16-06/23 | 483 |
| Zürich, Switzerland | 01/15-12/22 | 1’183 |
| **Total** |  | 13’525 |

| **Supplementary Table 3.** Full models of the main analysis with all variables. Odds ratios and 95%-confidence interval boundaries are rounded to 2 digits. If the rounded value is 1, we denote values smaller than 1 with (<1) and values larger than 1 with (1>). | | | | |
| --- | --- | --- | --- | --- |
| **Variable** | **Odds Ratio** | **Lower 95%-CI** | **Upper 95%-CI** | **p-value** |
| **Poor outcome at 3 months in survivors** | | | | |
| (Intercept) | 0.01 | 0.01 | 0.02 | <0.001 |
| Prestroke Dependency (Yes) | 0.68 | 0.57 | 0.81 | <0.001 |
| Age (per year) | 1.03 | 1.03 | 1.04 | <0.001 |
| Sex (M) | 0.92 | 0.84 | 1(>1) | 0.06 |
| NIHSS at Admission (per point) | 1.09 | 1.09 | 1.1 | <0.001 |
| EVT Complications (Yes) | 1.34 | 1.16 | 1.55 | <0.001 |
| Unsuccessful Recanalization (Yes) | 2.18 | 1.95 | 2.43 | <0.001 |
| Bridging (Yes) | 0.69 | 0.63 | 0.76 | <0.001 |
| Admission Glucose (per mmol) | 1.08 | 1.06 | 1.1 | <0.001 |
| Atrial Fibrillation (Yes) | 0.98 | 0.89 | 1.08 | 0.63 |
| Prior Ischemic Stroke (Yes) | 1.18 | 1.04 | 1.35 | 0.01 |
| Time to Groin (per 30min) | 1.01 | 1(>1) | 1.01 | <0.001 |
| **Death at 3 month** | | | | |
| (Intercept) | 0 | 0 | 0 | <0.001 |
| Prestroke Dependency (Yes) | 2.33 | 2.04 | 2.66 | <0.001 |
| Age (per year) | 1.05 | 1.05 | 1.06 | <0.001 |
| Sex (M) | 1.1 | 1(>1) | 1.21 | 0.05 |
| NIHSS at Admission (per point) | 1.09 | 1.08 | 1.1 | <0.001 |
| EVT Complications (Yes) | 1.79 | 1.55 | 2.07 | <0.001 |
| Unsuccessful Recanalization (Yes) | 1.79 | 1.61 | 1.99 | <0.001 |
| Bridging (Yes) | 0.63 | 0.57 | 0.7 | <0.001 |
| Admission Glucose (per mmol) | 1.07 | 1.06 | 1.09 | <0.001 |
| Atrial Fibrillation (Yes) | 0.85 | 0.77 | 0.94 | 0.001 |
| Prior Ischemic Stroke (Yes) | 1.19 | 1.05 | 1.35 | 0.01 |
| Time to Groin (per 30min) | 1(>1) | 1(>1) | 1.01 | 0.04 |
| **Symptomatic intracranial hemorrhage** | | | | |
| (Intercept) | 0.01 | 0.01 | 0.02 | <0.001 |
| Prestroke Dependency (Yes) | 0.72 | 0.53 | 0.98 | 0.04 |
| Age (per year) | 1(>1) | 1 (<1) | 1.01 | 0.46 |
| Sex (M) | 1(>1) | 0.84 | 1.19 | 0.98 |
| NIHSS at Admission (per point) | 1.03 | 1.01 | 1.04 | <0.001 |
| EVT Complications (Yes) | 3.76 | 3.1 | 4.55 | <0.001 |
| Bridging (Yes) | 1.18 | 0.99 | 1.4 | 0.07 |
| Admission Systolic Blood pressure (per 10 mmHg) | 1.03 | 1(>1) | 1.06 | 0.05 |
| Time to Groin (per 30min) | 1 (<1) | 0.99 | 1(>1) | 0.39 |
| **Any intracranial hemorrhage** |  |  |  |  |
| (Intercept) | 0.13 | 0.1 | 0.18 | <0.001 |
| Prestroke Dependency (Yes) | 0.88 | 0.77 | 1.02 | 0.09 |
| Age (per year) | 1 (<1) | 1 (<1) | 1(>1) | 0.45 |
| Sex (M) | 1.09 | 1 (<1) | 1.19 | 0.05 |
| NIHSS at Admission (per point) | 1.04 | 1.03 | 1.04 | <0.001 |
| EVT Complications (Yes) | 1.8 | 1.6 | 2.02 | <0.001 |
| Bridging (Yes) | 0.98 | 0.9 | 1.06 | 0.58 |
| Admission Systolic Blood pressure (per 10 mmHg) | 1.03 | 1.02 | 1.05 | <0.001 |
| Time to Groin (per 30min) | 1(>1) | 1 (<1) | 1(>1) | 0.67 |
| **Unsuccessful Recanalization** | | | | |
| (Intercept) | 0.31 | 0.23 | 0.43 | <0.001 |
| Prestroke Dependency (Yes) | 1.16 | 1.02 | 1.33 | 0.03 |
| Age (per year) | 1.01 | 1.01 | 1.01 | <0.001 |
| Sex (M) | 1.05 | 0.96 | 1.14 | 0.28 |
| NIHSS at Admission (per point) | 1.01 | 1 (<1) | 1.01 | 0.08 |
| EVT General anesthesia (Yes) | 0.95 | 0.87 | 1.03 | 0.22 |
| Bridging (Yes) | 0.97 | 0.89 | 1.06 | 0.52 |
| Admission Systolic Blood pressure (per 10 mmHg) | 0.96 | 0.95 | 0.97 | <0.001 |
| Atrial Fibrillation (Yes) | 0.82 | 0.75 | 0.9 | <0.001 |
| Diabetes Mellitus (Yes) | 0.9 | 0.8 | 1 (<1) | 0.04 |
| Time to Groin (per 30min) | 1(>1) | 1(>1) | 1.01 | 0.01 |

**Supplementary Figure 1.** Results of main analysis and sensitivity analyses. Purple: complete case analysis, cyan: imputed datasets (MICE imputation) using simple models, green: imputed datasets using the full models (main analysis, see **Figure 3**), orange: imputed datasets using the inverse propensity score matching models.

**Supplementary Figure 2.** Balancing of covariates for propensity score matching before and after adjustment. A. Poor outcome in survivors. B. Death at 3 months. C. Intracranial hemorrhage. D. Unsuccessful recanalization. Dotted line indicates 0.05.
